# Systematic identification of immunodominant CD4^+^ T cell responses to HpaA in *Helicobacter pylori* infected individuals

**DOI:** 10.18632/oncotarget.11092

**Published:** 2016-08-05

**Authors:** Jian Hu, Li Chen, Wuchen Yang, Bin Li, Heqiang Sun, Shanshan Wei, Yafei He, Zhuo Zhao, Shiming Yang, Quanming Zou, Weisan Chen, Hong Guo, Chao Wu

**Affiliations:** ^1^ Department of Gastroenterology, The Second Affiliated Hospital, Third Military Medical University, Chongqing, PR China; ^2^ Department of Intensive Care Unit, Chengdu Military General Hospital, Chengdu, PR China; ^3^ National Engineering Research Center of Immunological Products, Department of Microbiology and Biochemical Pharmacy, College of Pharmacy, Third Military Medical University, Chongqing, PR China; ^4^ Department of Blood Transfusion, The Second Affiliated Hospital, Third Military Medical University, Chongqing, PR China; ^5^ Department of Hematology, The Second Affiliated Hospital, Third Military Medical University, Chongqing, PR China; ^6^ T cell Laboratory, La Trobe Institute for Molecular Science, School of Molecular Science, La Trobe University, Bundoora, Victoria, Australia

**Keywords:** *Helicobacter pylori*, HpaA, immunodominant epitope, HLA-DRB1*0901, Immunology and Microbiology Section, Immune response, Immunity

## Abstract

In mice, antigen-specific CD4^+^ T cell response is indispensible for the protective immunity against *Helicobacter pylori* (*H. pylori*). It has been demonstrated that neuraminyllactose-binding hemagglutinin (HpaA) immunization protected mice from *H. pylori* infection in a CD4^+^ T cell dependent manner. However, much remains unclear concerning the human CD4^+^ T cell responses to HpaA. We conducted a systematic study here to explore the immunodominant, HpaA-specific CD4^+^ T cell responses in *H. pylori* infected individuals. We found that HpaA-specific CD4^+^ T cell responses varied remarkably in their magnitude and had broad epitope-specificity. Importantly, the main responses focused on two regions: HpaA76-105 and HpaA130-159. The HLA-DRB1*0901 restricted HpaA142-159 specific CD4^+^ T cell response was the most immunodominant response at a population level. The immunodominant epitope HpaA142-159 was naturally presented and highly conserved. We also demonstrated that it was not the broad peptide specificity, but the strength of HpaA specific CD4^+^ T cell responses associated with gastric diseases potentially caused by *H. pylori* infection. Such investigation will aid development of novel vaccines against *H. pylori* infection.

## INTRODUCTION

*Helicobacter pylori* (*H. pylori*) infects more than 50% global population [1]. The infection is closely associated with many gastric disorders, such as gastritis and peptic ulcers, and is the major cause of gastric cancer [2] that lead to more than half million deaths worldwide [3]. *H. pylori* infection is also the leading cause of antibiotic resistance that threatens the effective prevention and treatment of an ever-increasing range of infections caused by bacteria [4]. Development of effective vaccines against *H. pylori* infection is considered highly significant globally. However, this has been significantly hindered by our limited understanding of the protective immunity against *H. pylori*.

Studies in both rhesus macaques [5] and humans [6] showed that CD4^+^ T cells infiltrated in situ during *H. pylori* infection. Studies in mice further demonstrated that either vaccine-induced or infection-induced protective immunity to *H. pylori* relies on a strong CD4^+^ T cell response [7–9]. Previous studies have identified major antigens of *H. pylori* [10–12] targeted by specific antibodies. However, studies on *H. pylori*-specific CD4^+^ T cells mainly focused on the whole bacterial lysates and largely ignored individual antigens. More importantly, none of these studies led to the identification of *H. pylori*-specific CD4^+^ T cell epitopes [13–15].

Neuraminyllactose-binding hemagglutinin (HpaA) is reported as a protective antigen and potentially an excellent vaccine candidate due to its highly conserved sequence [16] and indispensable role for *H. pylori* colonization [17]. Oral immunization with recombinant HpaA protein protected mice from *H. pylori* infection and the protection was strongly associated with HpaA-specific mucosal CD4^+^ T cell responses [18, 19]. However, little is known about the human CD4^+^ T cell responses to HpaA in *H. pylori* infected individuals. Using a systematic approach, we have identified and characterized several immunodominant CD4^+^ T cell responses specific to HpaA in our previous study [20]. The immunodominant CD4^+^ T cell responses specific to HpaA88-100 were observed in most *H. pylori* infected individuals who expressed HLA-DRB1*1501 and were significantly more abundant in patients with less severe gastric diseases [20]. However, HLA-DRB1*1501 is only expressed by up to 10% Chinese Han population (http://www.allelefrequencies.net/default.asp), the extent and magnitude of HpaA-specific CD4^+^ T cell responses and their roles in *H. pylori* infection associated diseases in the rest 90% Han population not expressing HLA-DRB1*1501 remain unknown.

The aim of this study was to evaluate HpaA-specific CD4^+^ T cell responses in *H. pylori* infected, HLA-DRB1*1501 negative individuals without knowledge of their other HLA alleles. Using *in vitro* HpaA-stimulated polyclonal T cell cultures and HpaA overlapping peptides, we comprehensively investigated the extent and magnitude of anti-HpaA CD4^+^ T cell responses in 101 *H. pylori* infected, HLA-DRB1*1501 negative individuals. Several immunodominant epitopes have been identified. HLA-DRB1*0901 restricted HpaA142-159 specific CD4^+^ T cell response was the most dominant one at the population level. The sequence of HpaA142-159 was highly conserved among all current 30 *H. pylori* strains in the database. This immunodominant epitope might be important in designing an effective *H. pylori* vaccine.

## RESULTS

### The magnitude of HpaA-specific CD4^+^ T cell responses in *H. pylori* infected individuals

**Figure 1:**
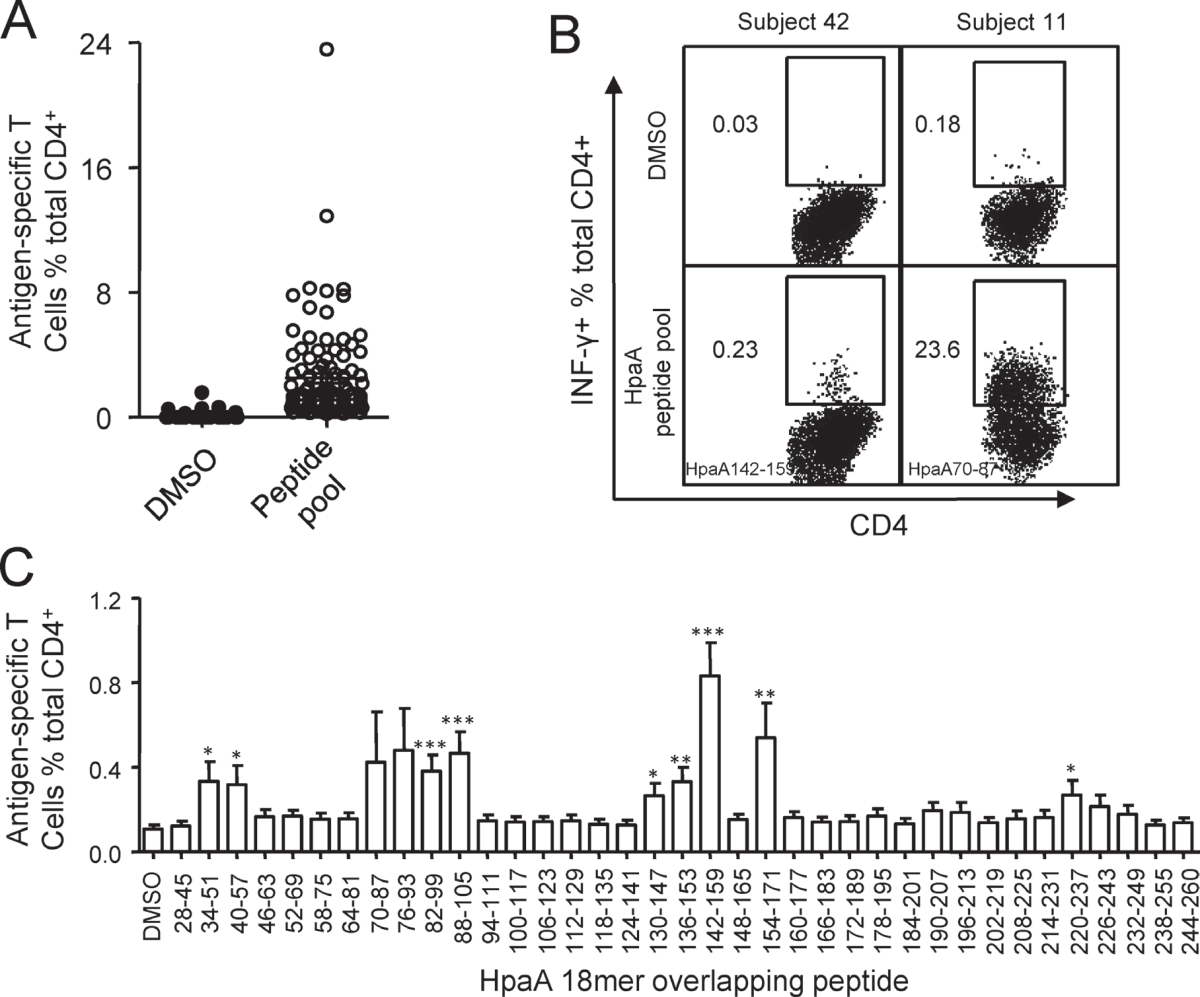
The magnitude of HpaA-specific CD4+ T cell responses in *H. pylori* infected individuals (**A**) PBMCs from 101 *H. pylori* infected subjects were stimulated with recombinant HpaA and the percentage of the IFN-γ producing antigen-specific CD4^+^ T cells were assessed using HpaA peptide pool dissolved in DMSO on day 13. DMSO was used as a control. The FACS plots of 2 representative subjects (subject 42 and 11) were shown in (**B**). The immunodominant 18mer peptides identified later were showed at the lower left quarter of the FACS plots. (**C**), the same HpaA-specific T cell cultures used in (A) were further screened for their specific response to the 37 overlapping 18mer HpaA peptides. The responses derived from all the 101 *H. pylori* infected subjects were compiled and statistically analyzed. Statistically significant differences between corresponding groups and DMSO control were determined by the Student *t* test: *, p<0.05; **, p<0.01; ***, p<0.001. The error bars indicate the standard error of the mean.

To quantify HpaA-specific CD4^+^ T cell responses in *H. pylori* infected individuals, polyclonal T cell cultures were expanded by stimulating PBMCs with recombinant HpaA protein as we previously reported [20]. The expansion procedure efficiently enlarged the frequency of HpaA-specific CD4^+^ T cells without priming antigen-unexperienced CD4^+^ T cells in the PBMCs collected from *H. pylori* uninfected subjects [20]. As shown in Figure 1A, HpaA-specific T cells were established using PBMCs from 101 *H. pylori* infected subjects and showed a Th1 cell phenotype [20]. The magnitude of these responses varied markedly (Figure 1B), the strongest response to the HpaA 18mer peptide pool was as high as 23.6% (subject 11) while the weakest response was only 0.23% (subject 42), and the average response was 2.5% approximately (Figure 1A). To better explore the magnitude and fine specificity of HpaA-specific CD4^+^ T cell responses, HpaA stimulated T cell cultures were further screened against 37 HpaA 18mer overlapping peptides individually. The strongest responses of single 18mer peptides were specific to HpaA70-87 derived from subject 11 (Figure 1B). When we averaged all detected responses of all 101 subjects, T cell response specific to HpaA142-159 was the strongest (Figure 1C); the remaining responses mainly focused on five regions covered by 8 peptides (HpaA34-51, HpaA40-57, HpaA82-99, HpaA88-105, HpaA130-147, HpaA136-153, HpaA154-171 and HpaA220-237), including the previously identified dominant response to HpaA88-100 in HLA-DRB1*1501 positive subjects (Figure 1C) [20].

### The relative strength of HpaA-specific CD4^+^ T cell responses in *H. pylori* infected individuals

**Figure 2:**
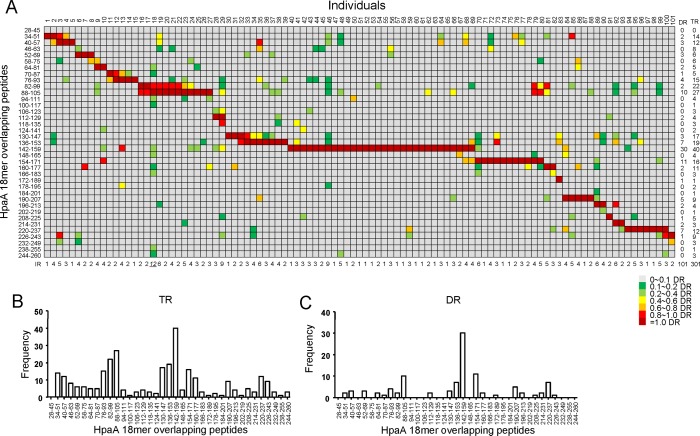
The relative strength of HpaA-specific CD4 T cell responses in H *pylori* infected individuals (**A**) The immunodominance hierarchies of all 101 subjects shown in Figure 1 were compiled as a “heat-map”. For each subject, the strongest response was considered as dominant response (DR) and was shown in dark red. All the other responses were evaluated by their relative strength against the dominant response. The responses were considered as non-specific when their strengths were not higher than 10% of their corresponding dominant response, and were marked in gray. The responses with strength between 10% and 99% of the dominant response were considered as subdominant ones and were assigned with a specific color in the heat-map. The number of Individual responses (IR), including dominant and subdominant ones in the same subject, were counted and shown below the graph. The number of Dominant responses (DR) and total responses (TR, including dominant and subdominant ones) specific to each 18mer-overlapping peptide were counted and shown on the right side the graph. The frequencies of the total responses (TR) and dominant responses (DR) were further analyzed against individual 18mer peptide and shown as bar charts in (**B**) and (**C**).

To better understand the fine immunodominance hierarchy of the HpaA-specific CD4^+^ T cell responses within these 101 individuals, all the HpaA-specific CD4^+^ T cell responses specific to each 18mer were summarized in Figure 2A as a “heat-map” according to their response intensity. In general a very broad epitope-specificity was identified, indicating that HpaA is indeed a highly immunogenic molecule. The fine specificity of these responses also varied remarkably, a result most likely dictated by the HLA polymorphism. However, most of the individual responses (IR, including dominant and subdominant responses in the same individual) recognized less than 4 peptides, 23 subjects had only a single HpaA epitope specific CD4^+^ T cell response detected, 25 subjects had two, 20 subjects had 3 and 17 subjects had 4. These results are in good agreement with the general immunodominance hierarchy for pathogen-specific cellular immunity [21]. There were also the odd exceptions, such as CD4^+^ T cells response from individual 18, had the broadest epitope specificity, 12 HpaA 18mer peptides were recognized. Overall, 301 responses were identified from these 101 subjects, almost three peptides were recognized on average in each subject, including neighboring peptides which sharing 12 aa of 18 aa.

The total responses (TR, including all of the dominant and subdominant responses) mainly focused on two regions (Figure 2B), they were HpaA76-105 and HpaA130-159, the same with the average responses as shown in Figure 1C. Studies from tumor antigen, such as NY-ESO-1, showed that the epitope distribution seemed to match hydrophobic regions better. However, it was not the case for HpaA as neither HpaA76-105 nor HpaA130-159 was located in hydrophobic regions (Supplementary Figure 1). Peptide HpaA142-159 specific CD4^+^ T cell responses were observed in 40 investigated subjects (Figure 2A and B), and were dominant in 30 of them (Figure 2A and C), representing the most immunodominant response to HpaA at the population level (Figure 2D).

### Fine characterization of the immunodominant CD4^+^ T cell response specific to HpaA142-159

**Figure 3:**
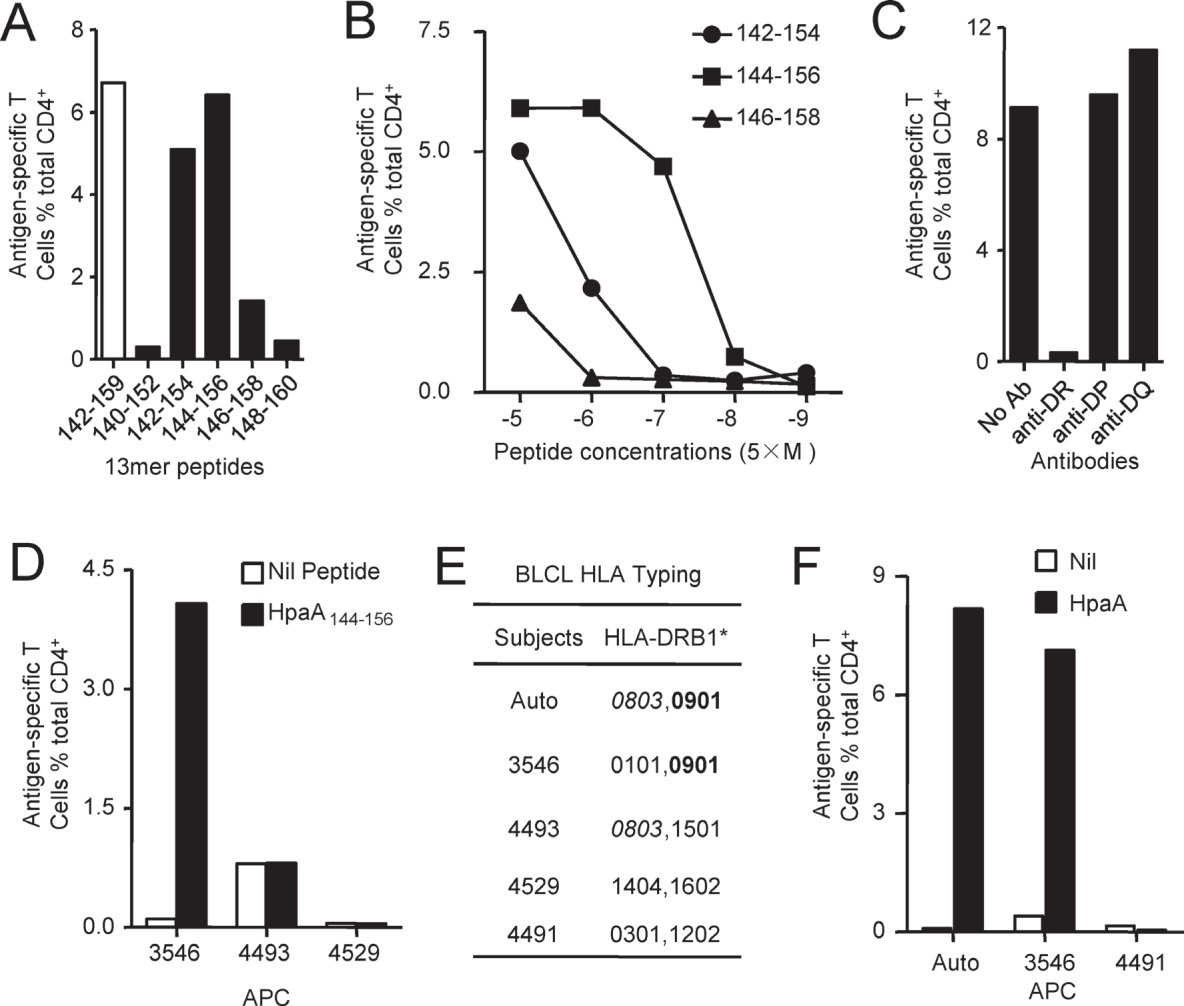
Fine characterization of the immunodominant CD4 T cell response specific to HpaA142-159 in individual 47 (**A**) The 13mer overlapping peptides within the HpaA142-159 18mer were screened, with the 18mer results being shown as open bar. (**B**) Three neighboring 13mer peptides were titrated to compare their activities for identifying the most potent core sequence. HLA class II antibodies (**C**) and partial HLA class II matched BLCLs (**D** and **E**) were used to identify the HLA allele presenting the HpaA144-156 13mer peptide. (**F**) Partial HLA class II matched BLCLs were pulsed with recombinant HpaA for 24 hours and then co-cultured with HpaA-specific T cells for 5 hours in the presence of monensin. IFN-γ-producing CD4^+^ T cells were determined by ICS to determine whether the epitope HpaA144-156 was a naturally processed peptide.

To further fine characterize the immunodominant CD4^+^ T cell response specific to HpaA142-159, screening of overlapping 13-mer peptides within the 18mer sequence was conducted to define the core sequence of the immunodominant epitope. As showing in Figure 3A, the immunodominant CD4^+^ T cells responding to the HpaA142-159 18mer peptide from individual 47 (Figure 2A) recognized three 13mer peptides. Among them, HpaA142-154 and HpaA144-156 stimulated comparable responses to that stimulated by the HpaA142-159 18mer peptide. Thus, peptide titrations were used to further confirm the epitope core sequence quantitatively. Titration of these three 13mers showed that HpaA144-156 was the most potent core epitope sequence (Figure 3B).

To determine the restricting HLA molecule for HpaA144-156, a class II antibody-blocking assay was conducted. The anti-DR antibody efficiently blocked T cell activation to peptide HpaA144-156, whereas the anti-DP and anti-DQ antibodies did not (Figure 3C). To further confirm the HLA-DR restriction of HpaA144-156, a panel of BLCLs with different DR alleles (Figure 3D and E) was used as APCs after being pulsed with HpaA144-156 to stimulate the peptide-specific T cell line. BLCL3546 expressing the same HLA-DRB1*0901 with autologous APC, efficiently activated peptide-specific T cells. In contrast, BLCLs 4493 and 4529, which did not express HLA-DRB1*0901, failed to present the peptide (Figure 3D and E). Therefore, the HpaA144-156-specific immunodominant CD4^+^ T cell response was restricted to HLADRB1*0901.

To determine whether this immunodominant epitope was indeed naturally presented by APCs, autologous BLCLs and other two BLCLs expressing different DR alleles (Figure 3E and F) were pulsed with recombinant HpaA for 24 hours and then co-cultured with HpaA144-156 specific T cells for 5 hours in the presence of monensin. ICS was performed to assess the ability of HpaA144-156 specific T cells to recognize these APCs. As shown in Figure 3F, autologous BLCLs and BLCL3546 expressing HLA-DRB1*0901 stimulated the HpaA144-156 specific T cells in an HpaA-dependent fashion. Thus, this immunodominant epitope HpaA144-156 could be naturally processed and presented by APCs.

### HpaA142-159 specific CD4^+^ T cell response was dominant in most of the HLA-DRB1*0901 positive individuals

**Figure 4:**
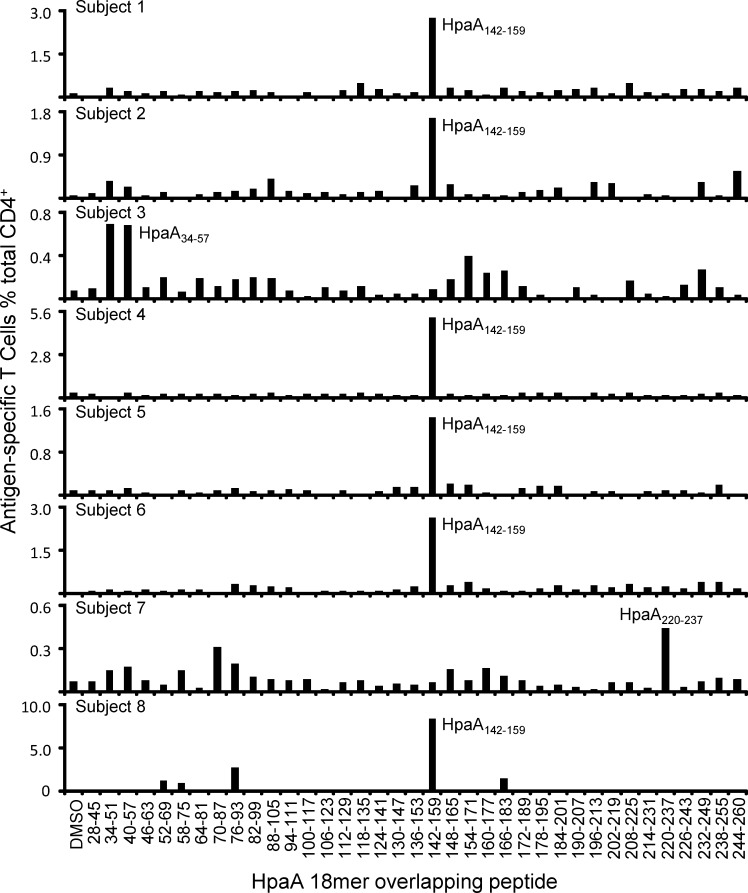
HpaA142-159 specific CD4 T cell response was dominant in most of the HLA-DRB1*0901-positive individuals HpaA-specific T cell cultures derived from 8 *H. pylori* infected subjects with HLA-DRB1*0901 expression were screened for their specific response to the 37 overlapping 18mer HpaA peptides in ICS assays.

We have previously demonstrated that HLA-DRB1*1501-restricted HpaA88-100 specific T cell responses were dominant in most of the HLA-DRB1*1501 positive individuals [20]. To confirm whether this also happened on the newly identified immunodominant epitope HpaA142-159, PBMCs from 8 HLA-DRB1*0901 positive subjects were stimulated by recombinant HpaA protein *in vitro*. Thirteen days later, the T cells were screened against 37 HpaA 18mer overlapping peptides. As showing in Figure 4, we found that 6 of the 8 HLA-DRB1*0901 expressing subjects (subject 1, 2, 4, 5, 6 and 8) had a dominant Th1 cell response specific to HpaA142-159, suggesting that HpaA142-159 specific CD4^+^ T cell response was dominant in most of the HLA-DRB1*0901 positive individuals.

### Associations of HpaA specific CD4^+^ T cell response with gastric diseases potentially caused by *H. pylori* infection

**Figure 5:**
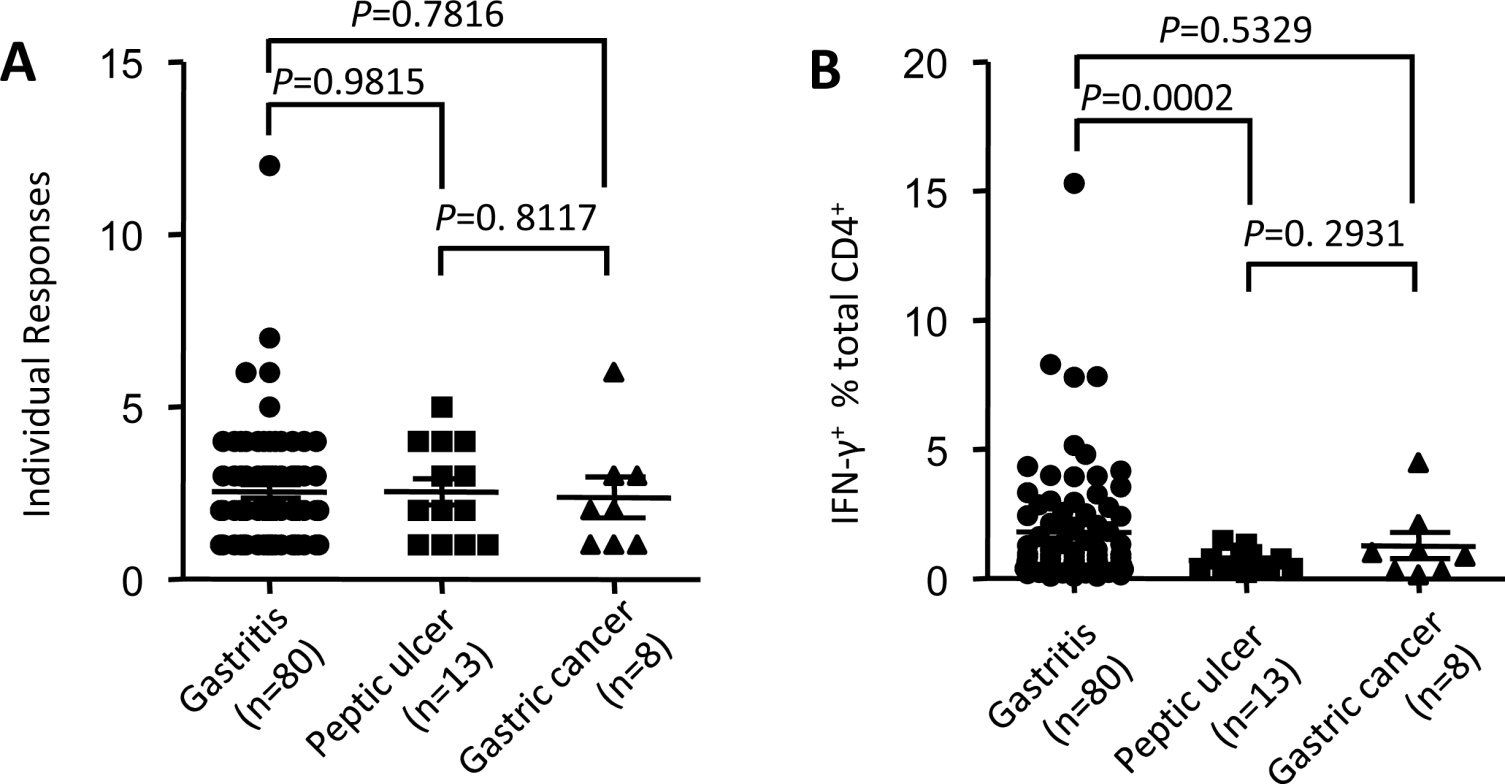
Associations of HpaA specific CD4 T cell response with gastric diseases potentially caused by *H. pylori* infection PBMCs from 101 *H. pylori* infected subjects with different gastric diseases were stimulated by recombinant HpaA protein in vitro. HpaA specific CD4^+^ T cell responses were assessed by ICS after 13 day's culture and the data analyzed according to gastric disease groups.

HLA-restricted CD4^+^ T cell responses were associated with HIV immune control [22] and disease activity of virulent Salmonella [23]. To verify whether such phenomenon existed during *H. pylori* infection, we evaluated the associations of HpaA specific CD4^+^ T cell response with gastric diseases potentially caused by *H. pylori* infection. As we showing in Figure 2A, the fine specificity of HpaA specific CD4^+^ T cell responses varied remarkably. However, no significant statistical difference of the number of individual responses were observed between gastritis, peptic ulcer and gastric cancer groups (Figure 5A), suggesting that the broad of peptide specificity had no associations with gastric diseases. Further comparisons showed that the percentage of HpaA specific CD4^+^ T cells in the gastritis group was significantly higher than that in the peptic ulcer group (Figure 5B), indicating that the strength of HpaA specific CD4^+^ T cells was associated with gastric disease potentially caused by *H. pylori* infection.

## DISCUSSION

Identification of a broad spectrum of immunodominant T cell epitopes across different HLAs is needed for the rational design of a T cell based vaccine against *H. pylori* [24]. However, only a few T cell epitopes derived from *H. pylori* antigens have been identified. As showing in Supplementary Table 1, a total of 20 epitopes are indexed in IEDB, including 5 mouse epitopes. Since the MHC molecules are highly polymorphic in mammals, MHC alleles of the same species generally select different peptides let alone MHC alleles from different species. For example, within the 11 reported CD4^+^ T cell epitopes derived from UreB antigen, not a single one is recognized by both murine and human CD4^+^ T cell responses (Supplementary Table 1). Similarly, CD4^+^ T cells of each subject showed various epitope specificities to even the same HpaA antigen (Figure 2A) as a result of MHC polymorphism. For the immunodominant epitope HpaA142-159, whether it could stimulate immunodominant CD4^+^ T cell responses in some other populations needs further investigation.

At the population level, almost all of the 18mer overlapping HpaA peptides stimulated positive CD4^+^ T cell responses (Figure 2A and B). However, in each individual, the response mainly focused on approximately three 18mer peptides on average (Figure 2A), consisting with the widely observed immunodominance phenomenon [25]. MHC alleles and T cell repertoire were considered as two most important determining factors of immunodominance [26, 27]. Data from the Allele Frequency Net Database (http://www.allelefrequencies.net/default.asp) show that the frequency of HLA-DRB1*1501 in the Chinese Han population is relative high (up to 10%). However, HLA-DRB1*0901 expressing individuals accounted for 28% in our study. HpaA88-100 specific CD4^+^ T cell response is immunodominant in HLA-DRB1*1501 expressing subjects [20]. Interestingly, HpaA142-159 specific CD4^+^ T cell response restricted to HLA-DRB1*0901 is the most immunodominant in an HLA-DRB1*1501 negative group (Figure 1C and Figure 2). Our data further confirmed the decisive role of MHC in immunodominance. As we failed to find any HLA-DRB1*1501 and HLA-DRB1*0901 double positive subjects with *H. pylori* infection, we could not evaluate which one of these two dominant responses was more immunodominant at individual level. In any case, a potential vaccine would have a broader coverage of population if both epitope sequences were included as vaccine candidate antigens.

Generally, immunodominant T cells are not only more prevalent, but also providing better protection than subdominant ones [28, 29]. The HpaA88-100 specific CD4^+^ T cell response was found to be associated with resistance to severe gastric diseases correlated to *H. pylori* infection in HLA-DRB1*1501 positive subjects [20]. A negative association with HLA-DRB1*1501 allele, reported in *H. pylori*-positive patients with gastric ulcers when compared with uninfected healthy controls, further supported this potential protective role mediated by HLA-DRB1*1501 restricted HpaA88-100 specific CD4^+^ T cell response [30, 31]. Here, we demonstrated that it was not the broad of peptide specificity, but the strength of HpaA specific CD4^+^ T cell responses was associated with gastric diseases potentially caused by *H. pylori* infection (Figure 5). HpaA specific CD4^+^ T cell responses were mainly observed in patients with gastritis, the mildest gastric disease associated with *H. pylori* infection, representing that HpaA specific CD4^+^ T cell response was protective (Figure 5). Studies in mice vaccinated with recombinant HpaA protein further supported this potential protective role mediated by HpaA specific CD4^+^ T cell responses [18, 19]. On the contrary, high seropositivity to HpaA has been associated with an increase risk for the development of gastric cancer [32], indicating that Th1-promoting adjuvant, such as oligonucleotides (CpG), would be better than Th2-promoting adjuvant when we use HpaA as the vaccine candidate antigen.

*H. pylori* has the ability to persist in the stomach and establish chronic infection. It is typically transmitted orally within families during early childhood and can persist for decades if no efficient eradication therapy was provided [33]. Chronic persistent infection gave the pathogen enough time to mutate and evade the host immunity, such as human immunodeficiency virus (HIV). However, unlike CD8^+^ T cell response, very limited evidence has shown that immunodominant CD4^+^ T cell response in *H. pylori* immunity could drive immune evasion. HpaA was considered as a potential vaccine candidate antigen since its highly conservative sequence [16]. Alignment of 11 HpaA epitope sequences in 30 *H. pylori* strains showed a few variants (Table 1). It would be interesting to see whether those epitope specific T cells could recognize their variants; how such mutations accumulated and what might be their influence on *H. pylori's* fitness. However, we noticed that the most immunodominant epitope HpaA142-159 was excellently conserved amongst these strains and only a single highly conserved substitution E to D was observed. This region might be critical for HpaA's adhesion function [17]. *H. pylori* might not be able to colonize in the stomach mucosal if HpaA142-159 mutated too much. Thus, such a conserved epitope peptide would be an ideal candidate antigen for a T cell based vaccine against *H. pylori* infection.

**Table 1:** Conservation of peptide sequences within 30 *H. pylori* strains

Peptide	Peptide sequence	Frequency (%) of peptide variants
HpaA34-51	ETNEVALKLNYHPASEKV	73.3
	*****T************	13.3
	*****T*******T****	3.3
	***********Q*****A	3.3
	*****S************	3.3
	*****************A	3.3
HpaA40-57	LKLNYHPASEKVQALDEK	90
	*******T**********	3.3
	*****Q*****A******	3.3
	*****************A	3.3
HpaA70-87	NIAKEYENKFKNQTALKV	66.7
	**************T***	26.7
	***************I**	3.3
	**************TI**	3.3
HpaA76-93	ENKFKNQTALKVEQILQN	66.7
	********T*********	13.3
	*********I********	3.3
	********T****E****	13.3
	********TI***E****	3.3
HpaA82-99	QTALKVEQILQNQGYKVI	66.7
	**T****E**********	13.3
	***I**************	3.3
	**T***************	13.3
	**TI***E**********	3.3
HpaA88-105	EQILQNQGYKVISVDSSD	33.3
	*E**********N*****	16.7
	************N*****	50
HpaA130-147	RPDPKRTIQKKSEPGLLF	90
	*******T**********	6.7
	*S****************	3.3
HpaA136-153	TIQKKSEPGLLFSTGLDK	93.3
	*T****************	6.7
HpaA142-159	EPGLLFSTGLDKMEGVLI	96.7
	*************D****	3.3
HpaA154-171	MEGVLIPAGFIKVTILEP	46.7
	**********V*******	50
	*D****************	3.3
HpaA220-237	IKSALNKIFANIMQEIDK	63.3
	**********S****M**	10
	**********S*******	16.7
	*********TS*******	3.3
	***V**************	6.7

HpaA sequences of 30 *H. pylori* strains available from the UniProt database were included, which were accessed on November 1, 2015. Only the 260 aa length HpaA sequences were selected.

In conclusion, we systematically evaluated the extent and magnitude of HpaA-specific CD4^+^ T cell responses in *H. pylori* infected individuals at a population level. The responses mainly focused on several regions, which were likely determined by the HLA genotypes of subjects investigated. HLA-DRB1*0901 restricted HpaA142-159 specific CD4^+^ T cell response was immunodominant in many of the 101 investigated subjects of Chinese Han population. Epitope HpaA142-159 showed high conservation and could be naturally processed and presented by antigen presenting cells. The peptide might be of important value for potential novel vaccines against *H. pylori*. It is also important to note that *H. pylori* encodes a large number of antigens and in some individuals the most dominant CD4^+^ T cell response may not be focused on HpaA. It is therefore very important to better understand T cell responses at a population level to other *H. pylori* encoded antigen and to identify other immunodominant T cell epitopes restricted by common HLA alleles. Once we know detailed cellular immunity to *H. pylori* a rational design of a T cell mediated vaccine against *H. pylori* will become possible.

## MATERIALS AND METHODS

### Subjects and blood samples

The study was approved by the Institutional Human Ethics Review Board of the Third Military Medical University. Participants were recruited from the Department of Gastroenterology at Xinqiao Hospital. They were diagnosed with gastric diseases by both endoscopic and histopathological diagnosis and screened for *H. pylori* infection status according to the Consensus definition of chronic gastritis in China - Second National Consensus Meeting on Chronic Gastritis [34] which is used in connection with the visual analog scale of the Sydney system [35]. Blood samples were collected with written informed consent. Peripheral blood mononuclear cells (PBMCs) were isolated by Ficoll-Hypaque (TBDscience, Tianjin, China) gradient and stored in liquid nitrogen until use. HLA typing was performed by PCR with sequencing-based-typing (PCR-SBT) at BGI (Shenzhen, china).

### Antigen, synthetic peptides and antibodies

Recombinant *H. pylori* HpaA was purified (purity >95%, data not shown) and stored at −70°C. 18mer peptides overlapping by 12 aa and 13mer peptides overlapping by 11 aa were synthesized and purified (purity >90%) by GL Biochem (Shanghai, China). All peptides were dissolved in DMSO (Sigma) and stored in −80°C. Anti-CD3 (PE), anti-CD4 (APC) and anti-IFN-γ (FITC) were purchased from Biolegend. Pan anti-DR (L243), anti-DP (B7/21) and anti-DQ (SPV-L3) antibodies were used as culture supernatants [36].

### Generation and culture of Epstein-Barr virus-transformed B lymphocyte cell lines (BLCLs)

BLCLs were established from autologous PBMCs by using the culture supernatant from EBV-producing B95-8 cells and cultured in “RP-10” consisting of RPMI-1640 (GIBCO) supplemented with 10% fetal calf serum (GIBCO), L-glutamine (2mM), 2-ME (5×10^−5^M) and antibiotics (penicillin 100 U/ml, streptomycin 100μg/ml).

### HpaA-specific T cell bulk culture

PBMC (1-2×10^6^) were pulsed with 0.2μM HpaA and cultured in 1 ml “RP-5” consisting of RPMI-1640 (GIBCO) supplemented with 5% human AB sera, L-glutamine (2mM), 2-mercaptoethanol (5×10^−5^M) and antibiotics (penicillin 100 U/ml, streptomycin 100μg/ml) in 48-well tissue culture plates. The medium was 50% replaced by “RP-5” containing 10U/ml recombinant human interleukin-2 (rhIL-2) on day 5 and then 50% replaced by “RP-5” containing 25U/ml rhIL-2 when required. Wherever possible replicated cultures were established based on the availability of PBMCs isolated from the patient blood samples.

### Intracellular cytokine staining (ICS)

In the 18mer and 13mer peptide screening assays, bulk cultured T cells were incubated with peptide at 5μM in “RP-5” at 37°C for 5h in the presence of monensin (Becton Dickinson). In assays assessing the restricting HLA alleles, BLCLs were pulsed with peptide of interest at 5μM for 1h, washed extensively and then co-cultured with bulk cultured T cells at a ratio of 1:10 for 5h in the presence of monensin. The cells were harvested, stained and analyzed by FACS as we described before [20]. In the antibody-blocking assay, APC were incubated with 10 μl of anti-HLA class II antibody supernatant for 30 min before addition of peptide and monensin. T cell activation was subsequently measured by ICS.[36]

### Bioinformatics analysis

Protein sequences were aligned and amino acid differences were scored to determine the sequence conservation of the newly identified dominant epitopes derived from HpaA within 30 *H. pylori* strains. The Universal Protein Resource (UniProt) database (http://www.uniprot.org/) was used (accessed on November 1, 2015) with the search criteria set as *Helicobacter pylori*, HpaA and full length (260aa) only, which identified 30 HpaA sequences from 30 completely different *H. pylori* strains. Protein sequences were aligned using the UniProt database, peptide regions were mapped, and frequency of mutation was determined across the various sequence groups.

### Statistical analysis

Unpaired t test was generally used to analyze the differences between two groups. However, when the variances differed, the unpaired t test with Welch's correction was used. Differences were considered significant when *P* value was less than 0.05. Correlations between parameters were assessed using the Pearson correlation analysis and linear regression analysis as appropriate.

## SUPPLEMENTARY MATERIALS


