# Supplementary material for: Systematic identification of immunodominant CD4+ T cell responses to HpaA in Helicobacter pylori infected individuals
Source: Oncotarget. 2016 Aug 5;7(34):54380–91. doi: 10.18632/oncotarget.11092 (PMC5342349; doi:10.18632/oncotarget.11092)
Supplement: Supplementary file 1 [file oncotarget-07-54380-s001.pdf]

Systematic identification of immunodominant CD4$^+$ T cell responses to HpaA in *Helicobacter pylori* infected individuals

Supplementary Material

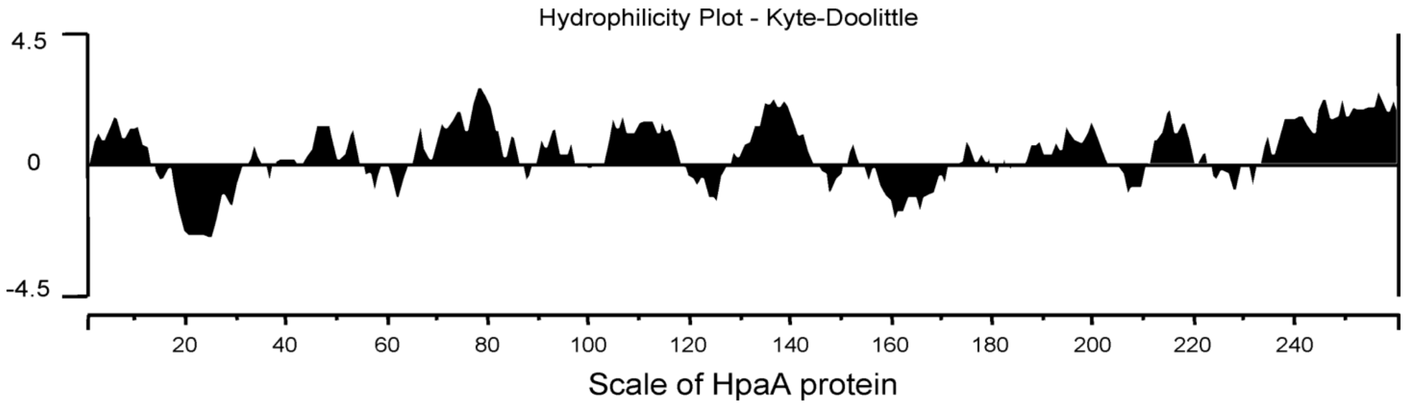

Supplementary Figure 1. Hydrophobicity analysis of HpaA.

Hydrophobicity analysis of HpaA protein was conducted by DNASTAR software.

Supplementary Table 1. *H. pylori* antigen derived $CD4^+$ T cell epitopes indexed in IEDB

Epitope name	Epitope sequence	MHC Restriction	Assay Method	Assay Readout	Reference
UreB(546-561)	FVDGKEVTSKPANKVS	H-2-IEd	3H-thymidine	proliferation	[1]
UreB(229-244)	SAINHALDVADKYDVQ	H-2-IAd	3H-thymidine	proliferation	[1]
UreB(237-251)	VADKYDVQVAIHTDT	H-2-IAd	3H-thymidine	proliferation	[1]
UreB(373-385)	ITRTWQTADKNKK	HLA-DRB1*14:04	ICS	IFNg release	[2]
UreB(373-390)	ITRTWQTADKNKKEFGRL	HLA-class II	ICS	IFNg release	[2]
UreB(403-420)	KRYLSKYTINPAIAHGIS	HLA-class II	ICS	IFNg release	[2]
UreB(438-452)	SPAFFGVKPNMIIKG	HLA-DRB1*08:03	ICS	IFNg release	[2]
UreB(439-456)	PAFFGVKPNMIIKGGFIA	HLA-class II	ICS	IFNg release	[2]
UreB(433-462)	DLVLWSPAFFGVKPNMIIKGGFIALSQMGD	HLA-class II	ICS	IFNg release	[2]
UreB(553-569)	TSKPANKVSLAQLFSIF	HLA-class II	ICS	IFNg release	[2]
UreB(321-339)	CHHLDKSIKEDVQFADSRI	class II	ELISA	IL-4 release	[3]
HpaA(88-100)	EQILQNQGYKVIS	HLA-DRB1*15:01	ELISPOT	IFNg release	[4]
HpaA(200-212)	SHSGGLVSTMVKG	HLA-DQB1*03:01	ICS	IFNg release	[4]
HpaA(192-204)	KFLKTTHSSHSGG	HLA-DRB1*04:06	ICS	IFNg release	[4]
MA(51-61)	SSHARPAFKGL	class II	3H-thymidine	proliferation	[5]
Pre-CR(115-129)	EEFIFHFIKNTRDGL	HLA-DRB1*16:02	ELISA	IL-2 release	[6]
Lpp20(83-97)	NQATAKARANLAANL	H-2-IAd	3H-thymidine	proliferation	[7]
Lpp20(58-72)	YEKYSGVFLGRAEDL	H-2-IAd	3H-thymidine	proliferation	[7]
UreA(183-203)	SVELIDIGGNRRIFGFNALVD	class II	ELISA	IL-4 release	[8]
UreA(74-90)	SHFHFFEVNRCLDFDRE	class II	biological activity	proliferation	[9]

UreA, Urease subunit alpha; UreB, Urease subunit beta; HpaA, Neuraminyllactose-binding hemagglutinin; Lpp20, lipoprotein Lpp20; Pre-CR, predicted coding region HP0964; MA, methionine aminopeptidase

Reference

1. Shi Y, Wu C, Zhou WY, Mao XH, Guo G and Zou QM. Identification of H-2d restricted Th epitopes in Urease B subunit of Helicobacter pylori. Vaccine. 2007; 25(14):2583-2590.
2. Yang WC, Chen L, Li HB, Li B, Hu J, Zhang JY, Yang SM, Zou QM, Guo H and Wu C. Identification of two novel immunodominant UreB CD4(+) T cell epitopes in Helicobacter pylori infected subjects. Vaccine. 2013; 31(8):1204-1209.
3. Guo L, Liu K, Zhao W, Li X, Li T, Tang F, Zhang R, Wu W and Xi T. Immunological features and efficacy of the reconstructed epitope vaccine CtUBE against Helicobacter pylori infection in BALB/c mice model. Applied microbiology and biotechnology. 2013; 97(6):2367-2378.
4. Chen L, Li B, Yang WC, He JL, Li NY, Hu J, He YF, Yu S, Zhao Z, Luo P, Zhang JY, Li HB, Zeng M, Lu DS, Li BS, Guo H, et al. A dominant CD4(+) T-cell response to Helicobacter pylori reduces risk for gastric disease in humans. Gastroenterology. 2013; 144(3):591-600.
5. Grogan JL, Kramer A, Nogai A, Dong L, Ohde M, Schneider-Mergener J and Kamradt T. Cross-reactivity of myelin basic protein-specific T cells with multiple microbial peptides: experimental autoimmune encephalomyelitis induction in TCR transgenic mice. Journal of immunology. 1999; 163(7):3764-3770.
6. Hansen BE, Rasmussen AH, Jakobsen BK, Ryder LP and Svejgaard A. Extraordinary cross-reactivity of an autoimmune T-cell receptor recognizing specific peptides both on autologous and on allogeneic HLA class II molecules. Tissue antigens. 2007; 70(1):42-52.
7. Li Y, Jiang Y, Xi Y, Zhang L, Luo J, He D, Zeng S and Ning Y. Identification and characterization of H-2d restricted CD4+ T cell epitopes on Lpp20 of Helicobacter pylori. BMC immunology. 2012; 13:68.
8. Guo L, Liu K, Xu G, Li X, Tu J, Tang F, Xing Y and Xi T. Prophylactic and therapeutic efficacy of the epitope vaccine CTB-UA against Helicobacter pylori infection in a BALB/c mice model. Applied microbiology and biotechnology. 2012;

95(6):1437-1444.
9. Guo L, Yin R, Liu K, Lv X, Li Y, Duan X, Chu Y, Xi T and Xing Y. Immunological features and efficacy of a multi-epitope vaccine CTB-UE against H. pylori in BALB/c mice model. Applied microbiology and biotechnology. 2014; 98(8):3495-3507.
